# Lactobacilli-Fermented Chia Seeds as a Potential Anti-Hypertensive Agent

**DOI:** 10.3390/molecules31142427

**Published:** 2026-07-10

**Authors:** Hector Atonal-Sánchez, Jorge Cornejo-Garrido, Flor N. Rivera-Orduña, Nemesio Villa-Ruano, Lidia Esmeralda García-Díaz, Maricruz Rangel-Galván, Silvia Luna-Suárez

**Affiliations:** 1 Centro de Investigación en Biotecnología Aplicada, Instituto Politécnico Nacional, Carr. Estatal Tecuexcomac-Tepetitla Km 1.5, Tepetitla 90700, Tlaxcala, Mexico; hecctorh2n2@gmail.com; 2 Laboratorio de Biología Celular y Productos Naturales, Escuela Nacional de Medicina y Homeopatía, Instituto Politécnico Nacional, Gustavo A. Madero, Mexico City 07320, Mexico; 3 Laboratorio de Ecología Microbiana, Departamento de Microbiología, Escuela Nacional de Ciencias Biológicas, Instituto Politécnico Nacional, Mexico City 07700, Mexico; friverao@ipn.mx; 4 SECIHTI-Dirección de Innovación y Transferencia de Conocimiento, Benemérita Universidad Autónoma de Puebla, Puebla 72570, Puebla, Mexico; nemesio.villa@secihti.mx (N.V.-R.); maricruz.rangel@correo.buap.mx (M.R.-G.); 5 Laboratorio de Adsorción y Cromatografía, Centro de Química Edificio IC7, Instituto de Ciencias, Ciudad Universitaria, BUAP. Av. San Claudio S/N, Puebla 72570, Puebla, Mexico; esmeralda.garcia@correo.buap.mx

**Keywords:** angiotensin converting enzyme, spontaneously hypertensive rats, fermented product, *Salvia hispanica*, lactic acid bacteria, proteolysis

## Abstract

Hypertension is a prevalent disorder that results in millions of deaths worldwide. In this regard, timely diagnosis and effective treatment are paramount for maintaining optimal blood pressure levels in the early stages of the disease. The objective of the present study was to synthesize and assess the effect of peptides derived from chia seeds that had undergone fermentation with *Lacticaseibacillus paracasei* strain 2501 (hereinafter referred to as LPDP, i.e., *L. paracasei*-derived products from this fermentation) on the angiotensin-converting enzyme (ACE) and the spontaneously hypertensive rat model (SHR). LPDP exhibited competitive inhibition, as evidenced by the identification of seven peptides in the <1 kDa fraction by HPLC-QToF-MS. The LPDP exhibited an IC_50_ of 11.1 μg/mL on ACE. The oral administration of 50 mg/kg body weight (BW) to SHR over a 14-day period resulted in a significant reduction in systolic pressure from 152 to 87 mmHg, accompanied by a substantial decrease in diastolic pressure from 117 to 74 mmHg. It is noteworthy that doses of 500 mg/kg BW led to a significant reduction in systolic pressure, from 154 to 68 mmHg and diastolic pressure, from 117 to 42 mmHg under identical experimental conditions. The hematological profiling of the assayed animals revealed that LPDP has no adverse effects at the cellular or biochemical level. These findings indicate the anti-hypertensive properties of LPDP and its possible application in the treatment of blood pressure.

## 1. Introduction

Hypertension is a prevalent chronic condition that is characterized by the persistent presence of elevated blood pressure levels (HBP). This condition is a major contributing factor to mortality rates that are high across the globe. Despite extensive research, no cure for HBP has been identified. Pharmacological and physical treatments are considered the most common alternative for controlling the disease [1]. One of the primary mechanisms by which blood pressure is regulated is through the modulation of the angiotensin-converting enzyme (ACE), which is involved in the renin–angiotensin–aldosterone system (RAAS). The enzyme in question catalyzes the transformation of the decapeptide angiotensin I into the octapeptide angiotensin II, a potent vasoconstrictor that plays a significant role in the regulation of blood pressure [2].

On the other hand, chia seeds (*Salvia hispanica* L., Lamiaceae) have gained significant attention due to their acknowledged health promoting properties [3]. These seeds are notable for their high content of polyunsaturated fatty acids, specifically α-linolenic acid, and docosahexaenoic acid, which have been demonstrated to enhance immune response, promote skin health, and offer myriad additional benefits [4]. It is also a significant source of minerals such as calcium, phosphorus, magnesium, potassium, iron, zinc and copper [5]. Chia seeds have been found to contain flavonoids such as quercetin and kaempferol, as well as polyphenols including chlorogenic acid [6]. Chia seeds have been identified as a significant source of protein and essential amino acids. In addition, various anti-diabetic, anti-oxidant, and anti-hypertensive compounds have been identified in this plant, suggesting its potential to prevent cardiovascular diseases [3]. Given the high protein content of chia seeds, fermentation processes using lactic acid bacteria to obtain bioactive peptides with anti-ACE activity can be regarded as a cost-effective method [7].

It is known that the proteolytic system of *Lactobacillus* spp. is capable of hydrolyzing proteins that have been recovered from the environment. Consequently, lactic acid bacteria (LAB) are frequently selected for the purpose of food fermentation and the production of antihypertensive peptides. The proteolytic system comprises cell envelope proteases, which play a crucial role in the process of protein breakdown. These proteases are responsible for the production of oligopeptides or smaller peptides that exhibit biological activity. The oligopeptide transport system is responsible for the transportation of these molecules to the surface of the bacterial membrane, whereas intracellular peptidases perform oligopeptide cleavage into smaller peptides or amino acids [8]. The activity of extracellular peptidases has been demonstrated to enhance these latter mechanisms [9]. Furthermore, the obtained peptides are transported by peptide transport systems, including the di- and tri-peptide transport systems driven by ATP hydrolysis [8]. Subsequent to absorption, peptides are utilized as nutrients by bacterial cells [10]. Lactic acid bacteria (LAB) are defined by their intricate nutritional needs, exhibiting auxotrophic characteristics for specific amino acids. Basic amino acids are obtained through the process of proteolysis and are utilized for bacterial development. In contrast, other non-degraded peptides exhibit distinct therapeutic properties [11]. In consideration of the aforementioned arguments, the present investigation was undertaken to examine the production of bioactive components from demucilaginated chia flour that had undergone fermentation by *Lacticaseibacillus paracasei.* The samples obtained were then subjected to a series of tests. These tests were designed to evaluate their impact on the enzymatic activity of lung rabbit ACE and the SHR model. The purpose of these tests was to substantiate their antihypertensive properties.

## 2. Results

### 2.1. Taxonomic Assignation

The 16s gene fragment was amplified using the primers 27F and 1492R, and the complete alignment of the sequence was analyzed with BLASTN version 2.2.18 online for nucleotide similarity against 16s rRNA. The results showed a percentage of 92% and an identity percentage of 96.86%. The species identified for this bacterium was *Lacticaseibacillus paracasei*, and the phylogenetic inference analysis with the 16s rRNA gene showed that the strain 2501 is distributed in a consistent clade with species of *Lacticaseibacillus*. To acquire further information, please refer to the Supplementary Materials.

### 2.2. Effect of Chia Medium Fermentation by Lacticaseibacillus sp. on the Bassis of Its Growth Stage

**Figure 1:**
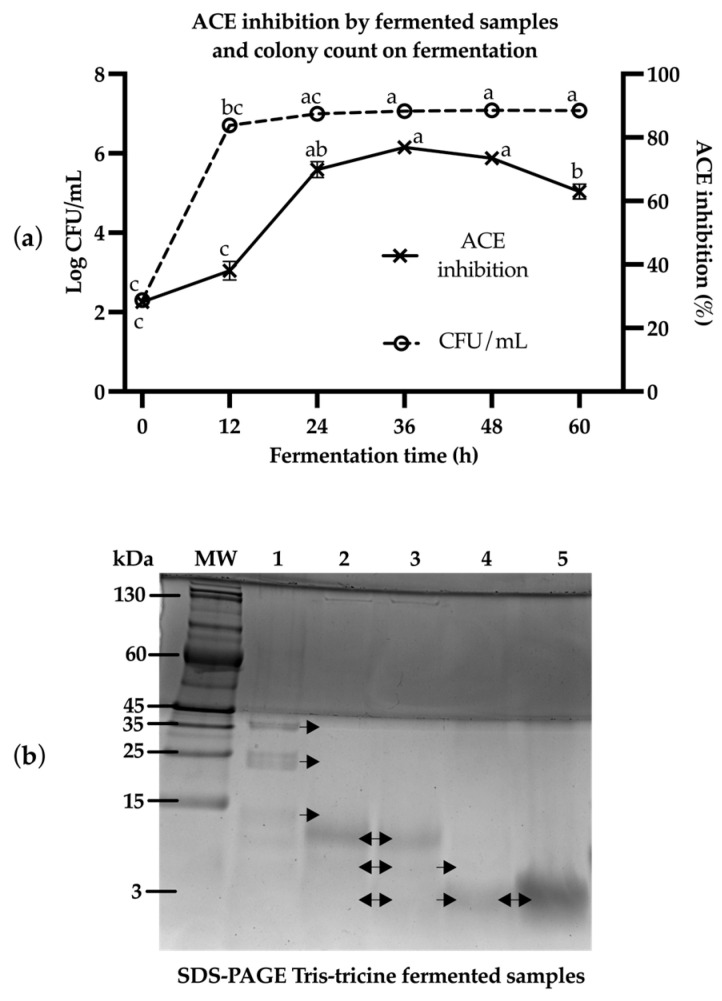
(**a**) Percentage of inhibition on ACE exerted by samples obtained as a product of fermentation, and colony count of the samples. All samples were tested at 0.02 mg/mL protein. The bars represent means ± SEM. Points with different letters indicate statistically significant differences among means determined by ANOVA and Bonferroni as *post hoc* tests according to the normality test, (*p* < 0.05) (*n* = 3). For the bacterial growth kinetics, Kruskal–Wallis and Dunn *post hoc* tests were performed according to the normality test, (*p* < 0.05), (*n* = 3). (**b**) Polyacrylamide Tris-tricine gel electrophoresis of fermentation samples. MW, molecular weight marker; 1, fermentation sample obtained at 12 h; 2, fermentation sample obtained at 24 h; 3, fermentation sample obtained at 36 h; 4, fermentation sample obtained at 48 h; 5, fermentation sample obtained at 60 h. Black arrows indicate marked changes in the peptide profiling.

A discernible shift in the degree of ACE inhibition was observed among the fermented samples obtained at varying times (Figure 1a). According to the findings of this study, no substantial changes in ACE inhibition (*p* < 0.05) were detected between 0 and 12 h. However, after 24 h, a notable surge in the percentage of inhibition was observed, reaching 69% compared to the level recorded at 12 h of fermentation. This peaked at 36 h and remained relatively stable (*p* < 0.05) until 48 h. Interestingly, the percentage of inhibition decreased to 62% after 60 h of fermentation. Concurrently, it was ascertained that the bacterium’s exponential growth phase occurs within the initial 12 h of fermentation. Subsequent to this, from 24 to 60 h, the stationary growth phase of the bacterium ensues. A discernible shift in the protein profile was evident in the examined samples, with these alterations exhibiting a direct correlation with the duration of fermentation (Figure 1b). Bands located in lane 1 of Figure 1b (between 35 and 25, and below 15 kilodaltons) were not detected in lane 2, whereas bands below the 15 kDa threshold were absent in the first lane, but are noted in lanes 2 and 3, and are lost in lanes 4 and 5. Interestingly, bands in the 3 kDa zone appeared in lane 2 and were maintained and intensified through lanes 3, 4 and 5.

### 2.3. Effect of LPDP on ACE Activity

**Figure 2:**
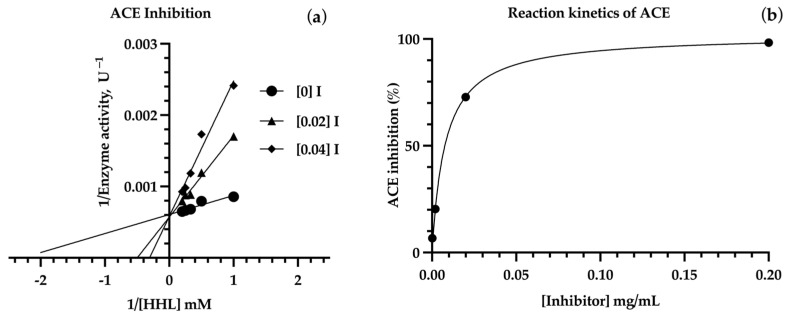
(**a**) Lineweaver-Burk plot representation for different concentrations of LPDP (0, 0.02, 0.04 mg protein/mL) on ACE activity. (**b**) Rectangular hyperbola graph for ACE inhibition at different inhibitor concentrations (0.2, 0.02, 0.002, 0.0002 mg/mL). All values are shown as means ± SEM (*n* = 3).

The inhibitory effect of different concentrations of LPDP on ACE resulted in a substantial increase in K_m_ (Figure 2a), indicating that a greater amount of substrate would be required to reach the maximum rate in the presence of an inhibitor in the reaction. The results demonstrated, as illustrated by the Lineweaver–Burk plot, that there were no alterations in maximum velocity in response to an increase in the concentration of the inhibitor. However, it was observed that a greater quantity of substrate was required for this effect to occur. To obtain the value of K_m_, a linear regression analysis was performed to fit the slope. In a similar manner, it was observed that the lines converge on the Y axis, indicating a competitive inhibition. As demonstrated in Figure 2b, an increase in inhibitor concentration results in a decrease in enzyme activity. This indicates that as the inhibitor concentration rises, the percentage of inhibition also increases, although the total inhibition is never attained. The IC_50_ was determined to be 11.1 µg/mL through the implementation of linear regression analysis on the data presented in Figure 2b.

### 2.4. Separation of the Fractions Obtained During Fermentation

**Figure 3:**
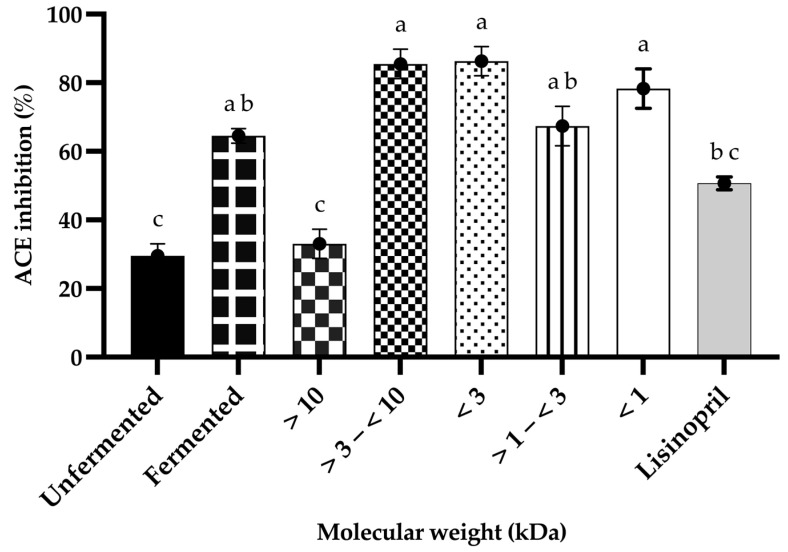
Percentage of inhibition of unfermented chia flour, fermented chia flour and ultrafiltration fractions on in vitro ACE. All samples were tested at 0.02 mg protein/mL. Lisinopril at 19.3 nM. Bars represent the mean value of each fraction ± SEM (*n* = 3). Bars with different letters are statistically different, analyzed with ANOVA and Bonferroni as *post hoc* tests according to the normality test, (*p* < 0.05).

A mixture was prepared from LPDP using the previously described methodology. The results are represented in Figure 3, which indicates that the unfermented sample only inhibited 30% of the ACE activity, and the unfractionated fermented sample of LPDP had an inhibition percentage of 64%. Once the mixture was divided, the fraction of LPDP exhibiting the lowest activity was greater than 10 kD, with 33% inhibition. However, the fractions demonstrating the highest percentage of inhibition were greater than 3–<10 kDa, with 85%, and less than 3 kDa, with 86%. The fraction with a molecular weight less than 1000 Da exhibited 78% inhibition, while the fraction with a molecular weight between 1000 and 3000 Da exhibited 67% inhibition. However, there was no statistically significant difference between these two fractions (*p* < 0.05).

### 2.5. HPLC-QToF-MS Analysis

**Figure 4:**
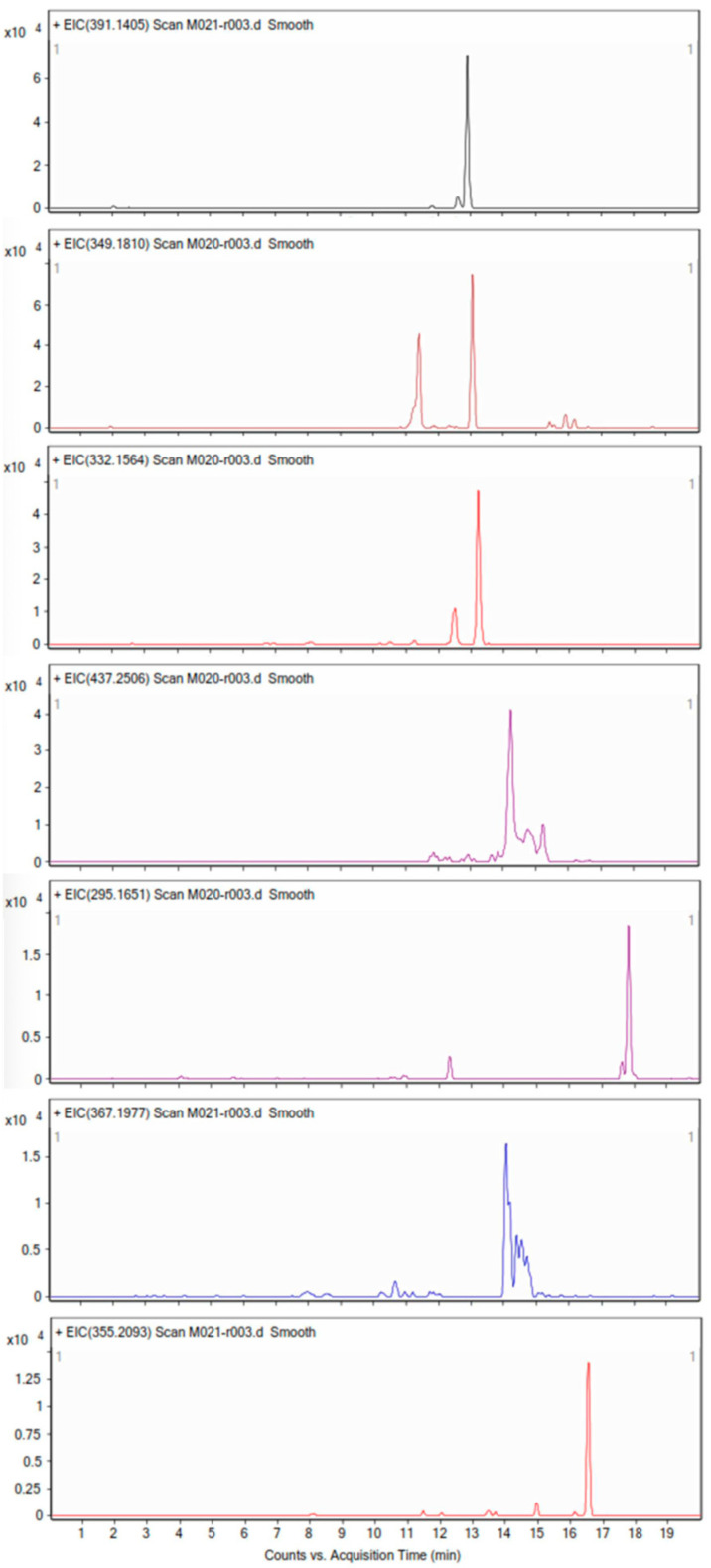
Results of the most abundant peaks of the chromatogram of the <1 kDa fraction of LPDP analyzed in HPLC-QToF-MS in order of counts per second. The chromatograms EIC were obtained with a mass accuracy of less than 10 ppm against the theoretical mass calculated.

For the recognition of active peptides of the <1 kDa fraction, High-Pressure Liquid Chromatography–Quadrupole Time of Flight–Mass Spectrometry (HPLC-QToF-MS) was used. Seven important peaks at different acquisition times were observed (Figure 4), ranging from 12.9 to 17.8 min. These constituted the most prevalent peaks within the chromatogram. Peptides were found to have a length ranging from two to five amino acids (see Table 1), with a mass ranging from 295.16 to 437.25 Da. The predominant amino acids identified in these peptides were proline and glycine, with the presence of basic, amidic, and aromatic amino acids also noted. The peptides found were generally polar, but some exhibited hydrophobic characteristics.

### 2.6. Molecular Docking of Peptides of LPDP on ACE

**Figure 5:**
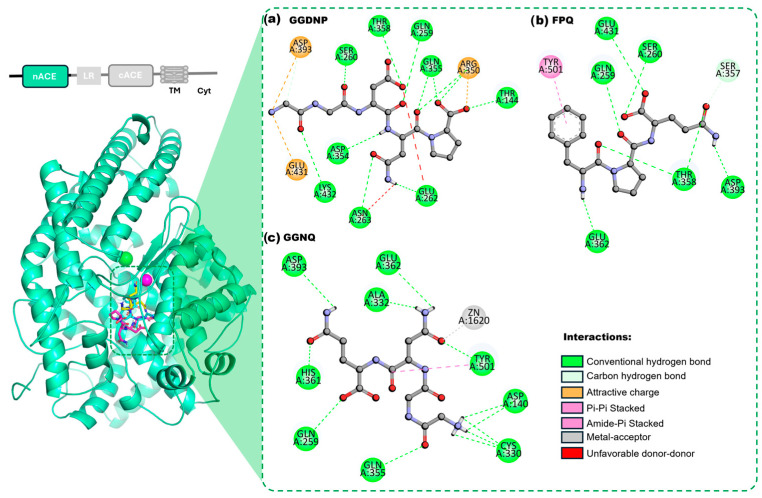
A three-dimensional diagram shows the nACE structure in complex with peptide inhibitors. The catalytic domains contain the Zn^2+^ and Cl^−^ ions, colored in magenta and green, respectively. The catalytic domains are connected by a short linker region (LR), subsequent bond with cACE subunit. next to a transmembrane (TM) region and a cytosolic (Cyt) domain. The close-up shows the two-dimensional diagram showing the main interactions of peptides: (**a**) GGDNP, (**b**) FPQ, and (**c**) GGNQ with the nACE subunit. Red circles indicate oxygen atoms.

**Figure 6:**
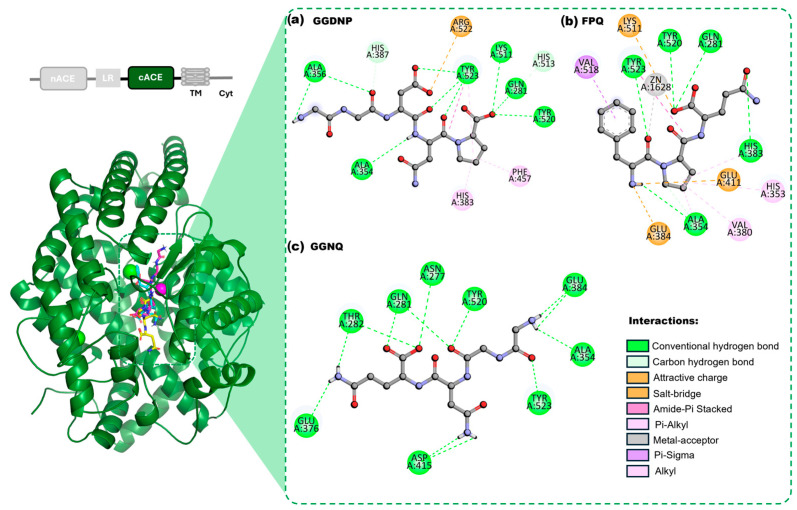
A three-dimensional diagram shows the cACE structure in complex with peptide inhibitors. The catalytic domains contain the Zn^2+^ and Cl^−^ ions, colored in magenta and green, respectively. The catalytic domains are connected by a short linker region (LR) with nACE subunit, and by a transmembrane (TM) region with the cytosolic (Cyt) domain. The close-up shows the two-dimensional diagram showing the main interactions of peptides: (**a**) GGDNP, (**b**) FPQ, and (**c**) GGNQ with the cACE subunit. Red circles indicate oxygen atoms.

The three peptides that were most abundant as determined by HPLC-QToF-MS were selected for computational evaluation. The docking analysis revealed differential binding tendencies of the evaluated peptides toward the human ACE catalytic domains. Among the tested ligands, GGDNP and FPQ displayed the most favorable binding energies in both nACE and cACE, whereas GGNQ consistently exhibited lower affinity values. In the nACE model, the optimal binding energies were approximately −8.76 kcal/mol for GGDNP and −8.65 kcal/mol for FPQ, while GGNQ exhibited weaker interactions of −7.71 kcal/mol. A similar trend was observed for cACE, where GGDNP achieved the strongest interaction energy of −8.93 kcal/mol, followed closely by FPQ at −8.82 kcal/mol, whereas GGNQ remained the least favorable peptide at −7.57 kcal/mol (see Table 2). The primary interaction residues of the GGDNP peptide within the nACE (Figure 5) active site involved the formation of multiple hydrogen bonds. It has been demonstrated that other binding formations include attractive charge interactions with D_393_, E_431_, and R_350_. For FPQ, the peptide’s primary interaction mechanism involved the formation of hydrogen bonds. Furthermore, a carbon–hydrogen bond was observed with S_357_, while a π-π stacked interaction was formed with Y_501_. In the cACE active site (see Figure 6), the GGDNP peptide formed a hydrogen bond. Additionally, carbon–hydrogen bond interactions were identified with H_387_ and H_513_, while an attractive charge interaction was observed with R_522_. It has been established that other binding formations include an amide–π stacked interaction with Y_523_ and π–alkyl interactions involving H_383_, F_457_, and Y_523_ residues. For FPQ, hydrogen bond formation was also observed. Furthermore, evidence of a carbon–hydrogen bond formation with A_354_ was observed, along with the detection of a metal-acceptor interaction involving Zn^2+^ 1628. A multitude of interactions have been observed, including attractive charge interactions with E_384_ and E_411_, a salt bridge with K_511_, a π-σ interaction with V_518_, an amide–π stacked interaction with Y_523_, and π–alkyl interactions involving V_380_ and H_353_. It has also been demonstrated that GGNQ primarily interacted through the formation of hydrogen bonds.

### 2.7. Antihypertensive Effect of LPDP in SHR

**Figure 7:**
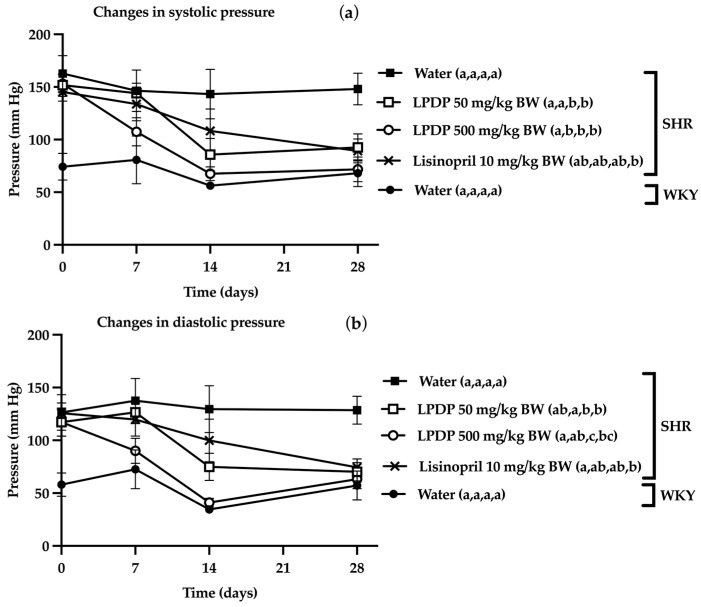
Changes in systolic (**a**) and diastolic pressure (**b**) after LPDP administration in SHR rats and monitored for 28 days. Sodium-free water was utilized as a negative control (SHR) and was also administered to the normotensive-WKY group. Lisinopril was used as a positive control at a dose of 10 mg/kg BW. The low and high doses of LPDP were 50 and 500 mg/kg BW in SHR groups respectively. The values represent the average value of each group ± SEM (*n* = 5). The legend with a different letter in parentheses for each group indicates the statistical difference between the points of each group, analyzed with ANOVA and Bonferroni as *post hoc* tests according to the normality test for SHR groups administered with LPDP and water, and Kruskal–Wallis and Dunn as *post hoc* tests according to the normality test for the rest of the groups (*p* < 0.05).

LPDP was given orally to SHR. The antihypertensive effect was evaluated by measuring blood pressure. Prior to the administration of the treatments, the blood pressures of all groups were monitored. Specifically, the SHR groups exhibited hypertension, while the Wistar Kyoto Rats (WKY) group demonstrated normotension. The normotensive control group depicted in Figure 7a exhibited a consistent mean systolic pressure value of 70 ± 13 mmHg and a mean diastolic pressure of 56 ± 12 mmHg. The hypertensive control group exhibited a mean systolic pressure value of 150 ± 18 mmHg and a mean diastolic pressure value of 130 ± 18 mmHg, respectively, without significant difference (*p* < 0.05) over the course of 28 days. A notable decrease in baseline systolic pressure values of 145 ± 9 mmHg was observed in the lisinopril group following 28 days of treatment, with final pressure values recorded at 89 ± 11 mmHg. A similar trend was observed in baseline diastolic pressure values, which decreased from 126 ± 10 mmHg to 74 ± 8 mmHg by the conclusion of the treatment period. In the group treated with 50 mg LPDP/kg BW, significant changes (*p* < 0.05) were also observed in the initial systolic pressure values of 152 ± 10 mmHg from 14 days of treatment, reducing the pressure to 87 ± 13 mmHg. This low pressure remained unchanged (*p* < 0.05) until the end of the experiment, as well as in the initial diastolic pressure values of 117 ± 13 mmHg and 74 ± 10 mmHg from 14 days of treatment. The most significant change (*p* < 0.05) was observed in the group treated with the high dose of LPDP, whose initial systolic pressure values were 154 ± 8 mmHg, reducing it to 107 ± 12 mmHg in only 7 days of treatment, but reaching 68 ± 12 mmHg from 14 days of treatment, and reaching 72 ± 12 mmHg at the end of the trial. In a similar manner, the initial diastolic pressure values depicted in Figure 7b underwent a reduction, from 117 ± 13 mmHg to 90 ± 12 mmHg at the seven-day mark, to 42 ± 4 mmHg at the 14-day stage of treatment. Ultimately, these values attained 63 ± 9 mmHg by the conclusion of the experimental period.

### 2.8. Effects of Administration of LPDP on SHR Blood Biometry

The majority of the hematological parameters obtained from the rats utilized in this experiment fell within the normal range (see Table 3). However, subtle variations were observed between the WKY and SHR strains in certain parameters, including mean corpuscular volume, average hemoglobin concentration, and mean corpuscular hemoglobin concentration. Nonetheless, these variations remain within the standard ranges reported for rats of their respective age and species. The platelet levels obtained from all groups were observed to be below the lower limit of normal. The proportion of basophils in all groups was at the upper limit of the normal range.

### 2.9. Effects of the LPDP Administration on the Biochemical Profile of SHR

The control group administered with lisinopril exhibited a marginally diminished discrepancy in serum glucose levels in comparison to the other groups. However, these values are within the range established as normal (Table 4) for this particular rat species and age. The creatinine levels of all groups were found to be elevated, with measurements indicating levels that are at the upper limit of the normal range. Conversely, the normotensive control group administered only with water of the WKY strain exhibited a modest variation in total cholesterol levels compared to the other groups, yet remained within the standard range for this parameter. The data obtained from high-density lipoprotein (HDL) and low-density lipoprotein (LDL) cholesterol in the WKY rat group exhibited a discrepancy when compared with the data from all SHR groups, which were situated in the lower zone but were all within the normal range. The level of direct bilirubin in the WKY group exhibited a discrepancy relative to the values of all the SHR groups, which were situated at the upper limit. The levels of alanine aminotransferase (TGP-ALT) and aspartate aminotransferase (TGO-AST) aminotransferase enzymes in the group administered with lisinopril were outside normal values, with a significant difference (*p* < 0.05) from the other groups. The glycated hemoglobin (HbA1c) values of all groups were at the upper limit of the normal range. The sodium levels in all of the groups were found to be at the lower end of the normal range.

## 3. Discussion

### 3.1. Proteolytic Activity of L. paracasei in Chia Medium Fermentation

In this study, *Lacticaseibacillus paracasei* was found to possess the capacity to hydrolyze the proteins present in the chia flour fermentation medium. This process resulted in the release of peptides that led to an increase in the percentage of angiotensin-converting enzyme (ACE) inhibition. This process occurs during the exponential phase of bacterial growth. In the present study, this phase was found to occur within the first 24 h, a finding that is consistent with the results reported by Liao et al. [12] in a recent study on the fermentation of milk with three different strains (*Lactobacillus delbrueckii* DPUL-F36, *Kluyveromyces marxianus* K1, K35, and *Lacticaseibacillus paracasei* DPUL-F115). The researchers’ findings indicated that the synergistic effect resulting from the concurrent presence of the three bacteria exhibited greater efficacy in inhibiting the angiotensin-converting enzyme (ACE) than the individual effects of each bacterium alone. This finding suggests that the concurrent activity of the three bacteria may facilitate the release of antihypertensive peptides. In addition, Kong et al. [13] reported alterations in the protein profile of pig sausage samples that were fermented with *Lactobacillus plantarum* following a 21-day period of incubation. In this regard, other researchers such as Sun et al. [14] have reported the proteolytic activity of *Lactobacillus* sp. CF80, which was observed to thrive in cereal crops such as tartary buckwheat. This is because lactic-acid bacteria (LAB) possess the capacity to hydrolyze the proteins present in the environment, thereby releasing bioactive peptides. They can also utilize complex carbohydrates, such as starch, without the necessity of supplementing additional carbon sources for growth.

In addition, a study conducted by Chen et al. [15] exhibited a comparable trend in terms of the percentage of ACE inhibition achieved following the fermentation of goat’s milk. It was observed that between 16 and 32 h of fermentation, a 70% inhibition of ACE was maintained. In the present study, the observed change in the electrophoretic pattern (see Figure 1b) and the percentage of inhibition (see Figure 1a) indicate a potential correlation between the extent of protein hydrolysis and the degree of ACE inhibition. The initial percentage of inhibition was 28%, which increased to 69% after 24 h. However, it decreased after 60 h. In a related study, Cui et al. [16] evaluated the effect of commercial enzymes on the degree of protein hydrolysis in milk. The findings indicated that the highest recorded inhibition percentage of 78% corresponded to a hydrolysis degree of 17%. A parallel outcome was identified in the research conducted by Pihlanto et al. [17], which indicated that the maximum percentage of protein hydrolysis occurred following 24 h of milk fermentation with *Lactobacillus acidophilus* ATCC 4356. The study further demonstrated that the optimal period for achieving the highest percentage of protein hydrolysis was between 24 and 44 h of fermentation, yielding an inhibition percentage ranging from 70% to 80%. However, it is important to note that continuous protein hydrolysis may have a deleterious effect on the inhibition of ACE. As Kunji et al. [18] reported, lactic acid bacteria have the capacity to hydrolyze certain proteins in order to obtain amino acids. This process would result in a loss of inhibitory activity due to the hydrolysis of active peptides.

### 3.2. The Effect of Fermented Chia Seeds on the Inhibition of Angiotensin-Converting Enzyme (ACE), a Critical Enzyme in the Renin-Angiotensin System

As illustrated in Figure 2a, the convergence point on the Y-axis is indicative of the maximum velocity (V_max_) of the reaction. This convergence point is determined by the reactions to different inhibitor concentrations. It is evident that V_max_ is reached when the substrate concentration is increased, thereby competing with the inhibitor for the enzyme’s active site. However, this finding also indicates that the LPDP-derived inhibitor should exhibit structural and functional characteristics analogous to those of the natural ACE substrate peptide (angiotensin I). In this natural substrate, the enzyme’s active site, comprising two histidines (His383 and His387) and glutamate (Glu411), forms a ligand with zinc. This process facilitates the formation of hydrogen bonds and hydrophobic interactions between amino acids from the ACE active site and those from the inhibitor [19]. As stated by Karatas et al. [20], this characteristic type of inhibition was mentioned in the parameters obtained from competitive inhibitors. Nonetheless, as illustrated in Figure 3, the purification or fractionation of the fermented sample is not required, as no substantial differences (*p* < 0.05) were observed in the percentage of inhibition between the unfractionated sample and the fractions. A parallel finding was documented by Lee et al. [21], who discovered that certain purified peptides of higher molecular weight exhibited an IC_50_ that was lower than that of peptides of lower molecular weight. A comparable outcome was attained in an eleven amino acid peptide (EGLPPRPKIPP), as documented by Geoffrey et al. [22]. It has been posited that, despite their lack of affinity for the active site of ACE, large peptides have the capacity to form hydrogen bonds and hydrophobic interactions through their C-terminal residues. This may result in substantial conformational alterations in the ligand and a subsequent inhibition of the angiotensin-converting enzyme (ACE).

### 3.3. Peptide Sequencing by HPLC-QToF-MS

These findings imply that the observed percentage of inhibition may be attributable to the size of the peptides, with smaller peptides exhibiting a greater inhibitory effect. The observed phenomenon may be attributed to their spatial conformation, which facilitates access to the active site. A thorough analysis by HPLC-QToF-MS revealed the presence of seven significant peaks. The peptide Lys-Pro-His-Pro exhibited the highest mass, measuring 437.25 Da. The hypothesis is that the presence of proline near the C-terminus in its sequence [23] contributes to the peptide’s potential for the greatest capacity to inhibit ACE. This phenomenon is also observed in the peptide Gly-Gly-Asp-Asn-Pro, which is the most abundant and contains proline. Given that cysteine-rich peptides are incapable of cleaving peptide bonds involving the amino group of a proline [24], it is conceivable that the peptide Phe-Pro-Gln may possess significant inhibitory capacity. According to the findings of previous studies, small peptides with a molecular weight of less than 1000 Da have been shown to possess a higher inhibitory capacity in comparison to peptides with a molecular weight greater than 1000 Da [25]. This analysis enabled the elucidation of the peptide sequence, a crucial step in comprehending its mechanism of action as an inhibitor of ACE. In silico analysis and peptide synthesis could facilitate the validation of peptides with inhibitory effects on ACE.

### 3.4. Analysis of the Interaction of LPDP Peptides Employing Molecular Docking Methodologies

The observed interaction patterns appear to be associated with the physicochemical properties of both the peptides and the ACE catalytic pockets. The negatively charged aspartic acid residue in GGDNP may favor electrostatic stabilization and hydrogen-bond formation, particularly within the relatively polar environment of nACE (Figure 5) [26]. In contrast, the aromatic and hydrophobic residues present in FPQ likely promote hydrophobic and π-type interactions within the more hydrophobic cACE catalytic cleft (Figure 6). Conversely, the absence of aromatic and strongly hydrophobic residues in GGNQ may contribute to its comparatively weaker interaction profile. Accordingly, both GGDNP and FPQ exhibited slightly more favorable interaction energies toward cACE. This finding suggests that hydrophobic stabilization may play an important role in peptide accommodation despite the overall structural similarity between ACE domains [27]. A similar set of interaction tendencies has been reported for a number of food-derived ACE inhibitory peptides that are enriched in hydrophobic, aromatic, and proline residues. These include LLIIPQH, LIIP, WWNW, WFRV, YYWK, WWDW, and WWTF. These peptides occupy the ACE catalytic gorge and interact with residues that are comparable to those identified in the present study [28,29]. Furthermore, the catalytic regions identified here partially overlap with binding environments described for inhibitory peptides such as FII, FPEQPP, VPP, and IPP. These include interactions near the Zn^2+^ associated with the HEXXH motif and polar networks involved in ACE domain selectivity [30,31].

### 3.5. Antihypertensive Effect of LPDP in SHR

The oral administration of 50 mg of LPDP/kg BW/day demonstrated a robust antihypertensive effect within a 14-day treatment period, resulting in a reduction in systolic pressure by 65 mmHg and diastolic pressure values by 43 mmHg. This outcome is analogous to the results obtained with lisinopril following 28 days of treatment at a dose of 10 mg/kg BW/day, which resulted in a decrease in systolic pressure of 55 mmHg and diastolic pressure of 51 mmHg. This finding suggests that the LPDP, administered at a dose of 50 mg/kg BW/day, exhibits a half-life that is half that of lisinopril, as illustrated in Figure 7a,b. In contrast, the highest antihypertensive effect was observed at a dose of 500 mg/Kg BW/day. In a period of seven days, systolic blood pressure demonstrated a significant decrease of 50 mmHg, while diastolic pressure exhibited a reduction of 31 mmHg. This decline is noteworthy, with a statistical significance determined by a *p*-value less than 0.05. This reduction in blood pressure is comparable to the effects observed with lisinopril, a pharmaceutical agent used to treat hypertension, when administered over a duration of 28 days. During this treatment period, the experimental group exhibited a reduction in systolic pressure of 80 mmHg and diastolic pressure of 56 mmHg. These outcomes approximated the systolic and diastolic pressure levels observed in the normotensive control group of WKY rats. Conversely, the SHR group that was administered sodium-free water exhibited elevated systolic and diastolic pressure values throughout the experimental period. In a similar manner, the systolic pressure values of the normotensive WKY group persisted at a low level throughout the experiment, a phenomenon that was concomitant with the maintenance of low diastolic pressure values.

The outcomes of this study demonstrated a superiority in effectiveness compared to the results reported by Toscano et al. [32]. The aforementioned research found that the incorporation of chia flour into the habitual dietary intake of individuals was inadequate to achieve a statistically significant reduction in blood pressure values (*p* < 0.05). The findings of this study are consistent with the results reported by Song et al. [33], who demonstrated that clams fermented with *Bacillus natto* led to a significant reduction in both the systolic and diastolic blood pressure of rats (*p* < 0.05).

The findings of the present experiment concerning the inhibition of angiotensin-converting enzyme (ACE) in vitro are consistent with those documented by Du et al. [34]. The researchers observed an increase in the percentage of ACE inhibition with an increase in the fermentation time of hydrolyzed fermented black sesame seeds by *Lactiplantibacillus plantarum*, ranging from 0 to 48 h. However, a notable decrease in inhibition was recorded at 60 h due to excessive hydrolysis caused by fermentation (*p* < 0.05). In a similar manner, Li et al. [35] observed that the inhibitory activity against ACE can be enhanced by 20% compared to the initial value through the fermentation of bacteria such as *Lactobacillus helveticus* H11, which are added to pasteurized commercial milk stored at 4 °C. Nevertheless, the attainment of this result required a duration of 21 days of fermentation.

### 3.6. Effect of LPDP Administration on the Hematological and Biochemical Parameters in SHR

As indicated by the findings presented in Table 3, the administration of LPDP in doses of 50 and 500 mg to SHR rats did not result in any observed alterations in hematological parameters. However, a platelet count lower than the normal range was observed in all groups, including those that were administered only with water. This phenomenon may be attributed to the higher propensity of rat blood to form platelet aggregations or hemolysis compared to human blood, as previously mentioned by Mamani et al. [36] in their study of blood and biochemical parameters in rats. It is imperative to acknowledge that each vivarium may possess its own reference values, given that there are factors such as breeding conditions, nutritional, energetic, or metabolic needs that each species and lineage may possess. This assertion is supported by the observations of values of the hematological and biochemical parameters of rats.

A review of the biochemical parameters presented in Table 4 reveals that the administration of LPDP in SHR rats did not result in any statistically significant differences (*p* < 0.05). However, a marginal increase in direct bilirubin levels was observed in the WKY group compared to the SHR groups. Sharma et al. [37] also allude to the potential for discrepancies in the levels of these parameters among different vivaria, attributable to factors such as geography, maintenance practices, and nutritional imbalances. An increase in transaminase levels was observed in the group administered with lisinopril. This phenomenon may be attributable to an uncommon adverse reaction. Although that has been reported, no such reactions have been observed in rats, including jaundice, nausea, or changes in urine color. Furthermore, there is no evidence that this is a dose-dependent problem, as previously mentioned by Sharma et al. [37] in their studies with lisinopril.

## 4. Materials and Methods

### 4.1. Mucilage Extraction Protocol

The removal of the mucilage from chia seeds is a prerequisite for successful handling, as this characteristic poses significant challenges during processing. The extraction of the mucilage gel was accomplished by hydrating the seeds with water at a ratio of 1:10 *w*/*v*. The mixture was stirred (Stuart® Roller mixer SRT6, London, UK) for a period of one hour. Subsequently, the mixture was subjected to a centrifugal process (Thermo Scientific® Legend XTR Centrifuge 6 × 250 mL, Waltham, MA, USA) at a speed of 15,344× *g* for a duration of 15 min at a temperature of 4 °C. The upper layer of the mixture, which primarily contained the mucilage, was decanted and subsequently measured. In parallel, the lower layer, which contained the seeds along with a smaller amount of water and mucilage, was weighed. This process yielded the ratio of water retained per gram of seed, thereby providing an estimation of the amount of mucilage present. Subsequently, the pellet was exposed to a drying process at a temperature of 50 °C (Rios Rocha® Drying Oven H41, Mexico City, Mexico). The dried seeds were subsequently sifted using a #20 mesh sieve (Mont inox® Test Sieve, Mexico City, Mexico). The process of rehydration of the dried seeds was performed under the same conditions for a second time.

The quantity of mucilage extracted was calculated as the amount of water retained following the second hydration in comparison to the initial hydration, expressed per gram of seed. This calculation was performed using the following formula:

Water Holding Capacity (WHC) = (Pellet − Initial Seed Weight)/Initial Seed Weight Percentage of mucilage removed = 100 − (A)

(A) = (100 × WHC2)/WHC1

The first stage of hydration and drying corresponds to WHC1. The seeds that are rehydrated and dried a second time correspond to WHC2.

### 4.2. Proximate Analysis of Demucilaginated Seeds

Subsequent to the removal of the mucilage from the seed, the seed was ground (CG, Electric Coffee Grinder, Model M150B, Zhongshan, China) with an electric mill until a homogeneous flour was obtained. Subsequently, a proximal analysis of the flour obtained was conducted. The moisture content was determined by following the AOAC 930.04 method of water loss by drying. The extraction of fat was accomplished by employing the Soxhlet technique (Lab Line Instruments, Soxhlet Extraction Model 5000, Melrose Park, IL, USA). The determination of the crude fat content was conducted in accordance with the AOAC method 920.39C. The total nitrogen content was determined by following the 955.04 method, as described by the AOAC, to ensure the accurate assessment of protein content.

### 4.3. Proteolytic Activity and ACE Inhibitory Activity

Three groups of bacteria were identified originally from an artisanal dairy product, based on their morphology and gram staining. These were isolated and cultivated in the M17 (Difco®, M17 Broth, Tucker, GA, USA) and MRS (Difco®, Lactobacilli MRS Broth, USA) medium. For the selection of strain, the organisms were inoculated in agar medium containing skimmed milk powder. The growth of these bacteria was meticulously monitored, and the formation of a halo of proteolytic activity was observed in two bacterial strains following 24 h of incubation at a temperature of 37 °C (Prendo Sev®, Incubator Model INO 650 V-9, Puebla, Mexico). Subsequently, two fermentations were performed, one for each bacterium, with a medium composed of demucilaginated chia flour suspended in water. The fermentation medium was sterilized prior to inoculation. A sample was extracted from the fermented medium, and the inhibitory activity of angiotensin-converting enzyme (ACE) (Sigma Aldrich, ACE From Rabbit Lung, CAS: 9015-82-1, Saint Louis, MO, USA) was subsequently measured to ascertain the optimal strain for the purpose of inhibition.

### 4.4. Bacterial Identification by 16S rRNA Gene

The bacterium, previously isolated and selected from the dairy product, was inoculated into MRS broth and subsequently incubated at 37 °C for 24 h. The bacterial DNA was extracted from a pure culture using the DNeasy Blood and Tissue Kit (QIAGEN, Germantown, MD, USA), in accordance with the manufacturer’s protocol. The 16s rRNA gene was amplified using the universal primers 27F and 1492R [38] (Lane 1991). The PCR amplifications were performed in a TC-5000 thermocycler (Techne, Staffordshire, UK) using a total reaction volume of 25 µL, which contained 50–100 ng of DNA template, 12.5 µL of PCR Master Mix (Ampliqon, Odense, Denmark), 0.1 µM of each primer, and 1 µL of GC enhancer (Applied Biosystems, Waltham, MA, USA). The reaction conditions comprised an initial denaturation step at 94 °C for 5 min, followed by 30 cycles at the same temperature for 5 min and 1 min, respectively. The annealing temperature was set to 57 °C for 1 min, followed by 72 °C for 1 min. The final extension step occurred at 72 °C for 5 min. Amplicon was sequenced at Macrogen Inc. (Seoul, Republic of Korea). A comparison was made between the sequence of strain 2501 and those deposited in GenBank and EzBioCloud databases. This sequence and other phylogenetically closely related species were aligned using MUSCLE (https://www.ebi.ac.uk/jdispatcher/msa/muscle) (accessed on 2 May 2026). Phylo-genetic analyses were performed using maximum likelihood on PhyML (http://atgc.lirmm.fr/phyml/) (accessed on 2 May 2026). The most suitable nucleotide substitution models were ascertained using the corrected Akaike Information Criterion (AICc) in jModelTest V2.1.10 Darriba et al. [39]. The nodes’ confidence was appraised through the implementation of a bootstrap test, which was executed following 1000 pseudo-replications. The sequence of *Lactobacillus acidophilus* (NR_043182) was used as the outgroup.

### 4.5. LAB-Facilitated Fermentation in Chia Medium and CFU/mL Count

The chosen strain was grown in an MRS liquid medium and used as a starter culture. The demucilaginated chia seeds were then ground into a flour, suspended in distilled water, and used as a fermentation medium at a ratio of 1:7.95 *w*/*v*. The fermentation medium was sterilized, inoculated with 1 mL of the chosen bacterium (OD = 0.9–1) (Thermo Fisher Scientific, Spectrophotometer Model Evolution 60s, USA) for 100 mL of medium, and incubated at 37 °C for 60 h. Samples were taken every 12 h, and a control of unfermented medium was maintained. The samples were stored at −20 °C until analysis. For the colony count (Stuart Scientific, Colony Counter, Model SC5, Cambridgeshire, UK), the Miles and Misra technique described by Khoda [40] was used. This method consists of performing serial dilutions from 100 to 10^−7^, or until a dilution that allows counting is reached. A sample of 0.02 mL of each dilution is taken and seeded in a solid medium on a plate. The plate is then left to incubate for 24 h at 37 °C. Counts are made for each dilution. The colony-forming unit count is calculated using the following formula:

CFU = number of colonies counted/[mL of sample inoculated × dilution factor reciprocal]

### 4.6. Separating Products of Fermentation by Ultrafiltration

After 24 h, the fermentation medium was centrifuged at 153× *g* for two minutes at 4 °C. The supernatant was separated and freeze-dried. A quantity of the lyophilized product was weighed and resuspended in distilled water at a ratio of 1:10 *w*/*v*. The freeze-dried, chia-fermented product (LPDP) was then centrifuged at 3836× *g* for 30 s at 4 °C. The phases were separated, and the supernatant was filtered for ultrafiltration using Amicon^®^ (Hailsham, UK) centrifuge filters with 10 and 3 kDa cut-offs, successively, at 3836× *g* for five minutes. Ultrafiltration discs from Merck (Boston, MA, USA) Millipore were then used for 1 kDa ultrafiltration.

### 4.7. LPDP Peptide Profile

An Agilent 1260 Infinity II HPLC was used to separate the previously fractionated sample (<1 kDa) with a QToF mass filter coupled to a mass spectrometer with an electrospray ionization (Agilent Technologies, ESI-L, low concentration tuning mix, G1969-85000, Waldbronn, Germany) source in positive polarity mode. The sample was treated with a 0.22 µm PTFE syringe filter prior to injection. The injected volume was 40 µL. The column was a Poroshell 120 EC-C18, 2.7 µm, 3 × 150 mm (Agilent, Santa Clara, CA, USA) at a temperature of 25 °C and a flow rate of 0.4 mL/min. The mobile phase was composed of A: water + 2 mM ammonium formate and B: acetonitrile + 2 mM ammonium formate. The gradient was 0% B from 0 to 5 min, 0–100% B from 5 to 15 min, and 0% B from 15.1 to 20 min. The fragmentation voltage was 175 V and the m/z range was 200–1000. Calibration was performed using ESI-L low conc. Tuning Mix G1969-85000, batch 0006776102 (Agilent Technologies, Waldbronn, Germany).

### 4.8. Computational Evaluation of Peptides as Potential ACE Inhibitors

Molecular docking calculations were performed using AutoDock Vina, version 1.2.5. The peptide ligands Gly-Gly-Asp-Asn-Pro (GGDNP), Phe-Pro-Gln (FPQ), and Gly-Gly-Asn-Gln (GGNQ) were generated in PyMOL v2.0. The peptide structures were corrected to include the appropriate N- and C-terminal protonation states corresponding to physiological pH conditions (pH 7.4). Then, the structures were optimized using Avogadro 1.2.0 [41]. The somatic human angiotensin-converting enzyme (ACE) domains were obtained from Protein Data Bank structures 2XYD (nACE) and 2XY9 (cACE). Both the receptor and the peptide structures were converted to PDBQT format using AutoDock Tools 1.5.7. Gasteiger charges were assigned and polar hydrogen atoms were added. During docking calculations, the receptor was treated as rigid, while the peptide ligands were considered flexible. Docking calculations focused on the catalytic gorge of ACE. The docking grid center was defined as x = 2.008, y = −16.819, and z = −21.649 Å for the nACE domain and as x = 14.452, y = −2.740, and z = −22.697 Å for the cACE domain. A grid box size of 30 × 30 × 30 Å was employed in both domains. Docking calculations were performed using an exhaustiveness value of 32, generating a maximum of 20 binding poses per simulation. The binding pose with the lowest binding free energy (ΔG) and the highest consistency among independent runs was selected as the most probable binding mode. Molecular interactions and binding orientations were visualized using PyMOL v2.0 and further analyzed using BIOVIA (San Diego, CA, USA) Discovery Studio Visualizer v20.1.0.19295.

### 4.9. Determination of ACE In Vitro Activity

The method described by Cushman & Cheung [42] with some modifications was used to measure ACE inhibition. In the first phase of the reaction, hippuryl-L-histidyl-L-leucine (HHL) (Sigma Aldrich, Hippuric Acid, CAS 495-69-2, USA) at a concentration of 5 mM was used as the substrate. Sodium tetraborate (Meyer®, Sodium Tetraborate, CAS 1330-43-4, Vallejo, CA, USA) at a concentration of 200 mM and sodium chloride (Meyer®, Sodium choride, CAS 7647-14-5, USA) at a concentration of 300 mM were used as the buffer. Lisinopril (Kener, Lisinopril, CAS 83915-83-7, Mexico City, Mexico) at a concentration of 19.43 nM was used as the positive control. ACE at an activity of 0.1 U/mL was used as the enzyme (Sigma Aldrich, ACE From Rabbit Lung, CAS: 9015-82-1, USA). Supernatant fermentation samples or LPDP were resuspended in distilled water at a concentration of 1:10 (*w*/*v*), and then the samples were centrifuged at 300× *g* for 30 s. The phases were separated, and a sample of the supernatant was taken and adjusted to a concentration of 0.02 mg of protein/mL. This sample was used as the inhibitor. In addition, a sample of the LPDP supernatant was separated by ultrafiltration and also used as the inhibitor. The reaction was incubated at 37 °C for 30 min and stopped by adding 1 M HCl. In the second phase, the quinoline-benzenesulfonyl chloride (BSC) (Sigma Aldrich, Quinoline, CAS 91-22-5, USA) (Sigma Aldrich, Benzenesulfonyl chloride, CAS 98-11-3, USA) chromogenic complex was added in the dark at a ratio of 3.2:1, and the mixture was incubated for 30 min at 30 °C. In the final phase, absolute ethanol (Hycel, Absolute Ethanol, CAS 64-17-5, Zapopan, Mexico) was added in the dark, and the mixture was incubated at 30 °C for 30 min. The hippuric acid reaction product released from HHL by ACE interacting with the quinoline-BSC solution was measured by UV-Vis spectrophotometry at 492 nm. The percentage of inhibition was calculated using the following formula

% ACE inhibition = [(A − B)/(A − C)] × 100

A = Uninhibited reaction.
B = Reaction with the presence of an inhibitor.
C = Sample with the presence of an enzyme inactivated with HCl.

### 4.10. Determination of Enzyme Inhibition Type

Enzyme activity was measured at different substrate concentrations [1,2,3,4,5] mM of HHL. The LPDP was resuspended in distilled water at a ratio of 1:10 (*w*/*v*) and centrifuged at 300× *g* for 30 s. The phases were separated and a sample of the supernatant was taken. This sample was adjusted to concentrations of [0, 0.02, 0.04] mg protein/mL and used as an inhibitor. The Lineweaver–Burk plot was used to represent the kinetics of the ACE reaction.

### 4.11. Polyacrylamide Gel Electrophoresis (SDS-PAGE) of Peptides in Tris-Tricine

The previously obtained LPDP was resuspended in distilled water at a ratio of 1:10 (*w*/*v*) and centrifuged at 300× *g* for 30 s. The phases were separated, and samples of the supernatant and an additional loading buffer (1.1:1) were taken. The loading buffer contained 2 mL of SDS at 10% (*w*/*v*). The mixture contained 1 mL of glycerol, 0.625 mL of Tris-HCl (1 M, pH 6.8), 6 mL of distilled water, and bromophenol blue dye. The supernatant mixture with charge buffer was then denatured with agitation at 95 °C for one minute (Eppendorf® Thermomixer Compact, Model 5350, Enfield, CT, USA). Twenty microliters of the sample were loaded into all lanes. The electrophoresis system described by Schägger [43] was chosen for this experiment. The separator gel contained 48% acrylamide and 3% bis-acrylamide (*w*/*v*). The spacer gel contained 48% acrylamide and 1.5% bis-acrylamide (*w*/*v*), and the stacking gel contained 30% acrylamide and 0.8% bis-acrylamide (*w*/*v*). The IRIS11 Prestained Protein Ladder was used as a molecular marker. A solution containing 0.5% Coomassie blue was used to stain the gel. These gels were run with a field strength of E = V/d 17.1 using the Mini PROTEAN System (Bio Rad, Mini PROTEAN Tetra Cell, Model 1658001, San Francisco, CA, USA).

### 4.12. Experimental Protocol Conducted in Rats

The present study utilized a total of 20 SHR rats and 5 WKY rats, with 5 rats from each group, at an age of 6 weeks and a body weight of 120 ± 8 g at the onset of the experiment. The rats were procured from the Instituto de Fisiología Celular at the Universidad Nacional Autónoma de México (UNAM). The rats were placed in plastic cages in groups and acclimatized for a period of two weeks prior to the initiation of the experiment. The experiment consisted of cycles of 12 h of darkness. The ambient temperature was measured at 23 °C. The rodent laboratory chow and water were provided ad libitum. The Bioethics Committee of the National School of Medicine and Homeopathy of the National Polytechnic Institute approved this research project (CBE/013/2025), which complies with International Standards and Policies.

The LPDP was resuspended in sodium-free water for administration in rats. Two doses of LPDP were evaluated during treatment: 50 and 500 mg LPDP/kg BW. Lisinopril at 10 mg/kg BW was utilized as a positive control (Kener, Lisinopril, CAS 83915-83-7, Mexico), while sodium-free water was employed as a negative control. The intragastric cannula was utilized for the administration of the test substance. The non-invasive tail cuff technique for measuring blood pressure was selected and evaluated using IITC Life Science’s MRBP rat equipment. The duration of the experiment was 28 days.

### 4.13. Blood Sampling for Hematological and Biochemical Analyses

All rodents were subjected to a 12 h fast prior to slaughter. A blood sample was obtained by cardiac puncture and placed in vacutainer tubes containing heparin. The tubes were then placed in a centrifuge at 1300× *g* for 10 min. The serum was separated to conduct a blood chemistry analysis, and an additional blood sample was used to perform blood counts. The samples were subjected to analysis in a designated clinical analysis laboratory.

### 4.14. Statistical Analysis

The analysis of the data from this project was conducted using GraphPad Prism version 10.3.1. The initial assessment entailed an examination for data cleansing, the identification of outliers, and a subsequent assessment of data normality. This approach enabled the determination of the most appropriate statistical analysis for each test. In order to ascertain any potential differences, an analysis of variance (ANOVA) was conducted. Subsequently, the Tukey, Bonferroni, or Kruskal–Wallis and Dunn post hoc tests were employed, depending on the distribution of the data or the number of multiple comparisons required. The data presented herein are expressed as the mean values ± standard error of the mean (SEM). The level of significance employed for the statistical tests was set at *p* < 0.05.

## 5. Conclusions

The findings of this study indicate that the strain utilized in this investigation, *Lacticaseibacillus paracasei*, exhibited the capacity to ferment a medium comprising demucilaginated chia flour. Furthermore, the fermentation process, spanning 24 h, resulted in the release of ACE-inhibiting molecules. It was also observed that this LPDP exerts competitive inhibition on this enzyme with an IC_50_ of 11.1 μg/mL. Conversely, the statistical analysis indicated that it is not necessary to separate or purify the LPDP to obtain the optimal percentage of inhibition for ACE, as the sample in its original state yielded a substantial percentage of inhibition. The peptides identified through HPLC-QToF-MS analysis suggest the presence of hydrophobic amino acids at the C-terminus. Previous studies have indicated that this feature contributes to enhanced inhibitory activity.

The computational evaluation of the selected peptides suggests that hydrophobic stabilization of GGDNP and FPQ peptides plays a significant role in peptide accommodation. Despite the overall structural similarity between ACE domains and the negative charge of GGDNP, aspartic acid enhances electrostatic stabilization and hydrogen bonding, primarily in the polar environment of nACE. In addition to the aforementioned observations, the in vivo trial revealed that a daily dose of 50 mg/kg BW of LPDP over a 28-day period is sufficient to reduce systolic and diastolic arterial pressure to 87/74 mmHg within 14 days, respectively. This effect is sustained with daily administration. A daily dosage of 500 milligrams per kilogram of body weight of LPDP is sufficient to reduce systolic and diastolic blood pressure, respectively, to 68/42 mm of mercury within a 14-day period of treatment. This effect is sustained with daily administration. A salient finding of the study is the absence of any alterations in the results of the hematological parameters and biochemical profile in any of the groups administered with LPDP. This finding suggests that it may be a safe and effective adjunctive treatment for blood pressure control. Further testing in clinical settings and exploration of the practical application of LPDP are necessary to validate these findings.

## Figures and Tables

**Table 1:** Identification of peptides of the <1 kDa fraction of LPDP analyzed in HPLC-QToF-MS.

Amino Acid Sequence	Counts per Second	Formula	Acquisition Time (min)	Mass (Da)
GGDNP	7.5 × 10^4^	C_14_H_22_N_4_O_9_	12.9	391.141
FPQ	7.5 × 10^4^	C_17_H_24_N_4_O_4_	13.1	349.181
GGNQ	4.9 × 10^4^	C_12_H_21_N_5_O_6_	13.2	332.156
KPHP	4.0 × 10^4^	C_20_H_32_N_6_O_5_	14.5	437.251
LY	1.9 × 10^4^	C_15_H_22_N_2_O_4_	17.8	295.165
KNF	1.7 × 10^4^	C_15_H_22_N_6_O_5_	14.1	367.197
RTH	1.4 × 10^4^	C_15_H_26_N_6_O_4_	16.6	355.209

**Table 2:** Molecular interactions between ACE inhibitor peptides and the ACE.

	nACE		cACE	
Peptides	Docked Energy (kcal/mol)	Interactions	Docked Energy (kcal/mol)	Interactions
GGDNP	−8.75	HB: T_144_, Q_259_, S_260_, N_263_, R_350_, Q_355_, T_358_, K_435_, D_354_, and E_262_; C-HB: D_393_; Attractive-charge: D_393_, E_431_, R_350_; U-DD: N_263_, Q_262_.	−8.93	HB: A_356_, A_354_, Y_523_, K_511_, Q_281_, Y_520_; C-HB: H_387_, H_513_; Attractive-charge: R_522_; Amide–π stacked: Y_523_; π–alkyl: H_383_, F_457_, Y_523_.
FPQ	−8.65	HB: E_362_, Q_259_, E_431_, S_260_, T_358_, D_393_; C-HB: S_357_; π-π stacked: Y_501_.	−8.8	HB: Y_523_, Y_520_, Q_281_, H_383_, A_354_; C-HB: A_354_; Metal-Acceptor: Zn 1628; Attractive-charge: E_384_, E_411_; Salt-bridge: K_511_; π-σ: V_518_; Amide–π stacked: Y_523_; π–alkyl: V_380_, H_353_.
GGNQ	−7.71	HB: D_393_, H_361_, Q_259_, E_362_, A_332_, Q_355_, Y_501_, D_140_, C_330_; Metal-Acceptor: Zn 1620; Amide–π stacked: Y_501_.	−7.57	HB: E_376_, T_282_, Q_281_, N_277_. Y_520_, E_384_, A_354_, Y_523_, D_415_.

**Table 3:** Blood cell parameters of SHR and WKY rats.

Parameter	Range	Strain	WKY	SHR			
		Group	Control Normotensive	Control (−)	Low Dose	High Dose	Control (+)
		Administration					
			Water	Water	LPDP (50 mg/kg BW)	LPDP (500 mg/kg BW)	Lisinopril (10 mg/kg BW)
Hemoglobin	14–18	Unit	15 ± 0.4 ^a^	14 ± 0.4 ^a^	15 ± 0.3 ^a^	14 ± 0.3 ^a^	14 ± 0.3 ^a^
		g/dL					
Hematocrit	40–52	%	44 ± 1.4 ^a^	42 ± 1.2 ^a^	43 ± 1.5 ^a^	42 ± 1.0 ^a^	42 ± 1.0 ^a^
Mean corpuscular volume (MCV)	49–58	fL(µm^3^)	54 ± 0.5 ^a^	51 ± 0.2 ^b^	51 ± 0.3 ^b^	50 ± 0.2 ^b^	50 ± 0.2 ^b^
Hemoglobin corpuscular mean (HCM)	17–20	pg	20 ± 0.07 ^a^	19 ± 0.06 ^b^	19 ± 0.1 ^b^	19 ± 0.09 ^b^	19 ± 0.1 ^b^
Mean corpuscular hemoglobin concentration (MCHC)	33–37	g/dL	36 ± 0.3 ^a^	37 ± 0.09 ^b^	38 ± 0.6 ^b^	37 ± 0.2 ^b^	38 ± 0.2 ^b^
Platelets	638–1177	µL	139 ± 38 ^a^	436 ± 119 ^a^	435 ± 123 ^a^	310 ± 84 ^a^	400 ±118 ^a^
Erythrocytes	7.3–8.8 (×10^6^)	µL	8 × 10^6^ ± 8 × 10^4 a^	8.3 × 10^6^ ± 2 × 10^5 a^	8.3 × 10^6^ ± 3 × 10^5 a^	8.5 × 10^6^ ± 2 × 10^5 a^	8 × 10^6^ ± 2 × 10^5 a^
Total leucocytes	6.6–20.3 (×10^3^)	µL	7.4 × 10^3^ ± 1 × 10^3 a^	6 × 10^3^ ± 1 × 10^3 a^	4.6 × 10^3^ ± 6 × 10^2 a^	4.7 × 10^3^ ± 6 × 10^2 a^	5.6 × 10^3^ ± 1 × 10^3 a^
Eosinophils	0.2–3.5	%	2.5 ± 0.6 ^a^	1 ± 0.6 ^a^	2 ± 0.4 ^a^	2 ± 0.3 ^a^	3 ± 0.2 ^a^
Basophils	0–0.8	%	1.5 ± 0.6 ^a^	0 ^a^	1 ± 0.3 ^a^	1 ± 0.4 ^a^	1 ± 0.3 ^a^
Lymphocytes	66–90	%	72 + 3 ^a^	85 + 2 ^a^	81 ± 3 ^a^	78 ± 4 ^a^	72 ± 11 ^a^
Monocytes	0.8–3.8	%	2 ± 0.3 ^a^	2 ± 0.5 ^a^	2 ± 0.6 ^a^	2 ± 0.7 ^a^	2 + 0.5 ^a^

The values shown represent the median of each group ± SEM (*n* = 5). Different letters displayed as superscripts in each row represent significant difference in each parameter between groups, analyzed with Kruskal–Wallis and Dunn as *post hoc* tests according to the normality test, (*p* < 0.05). The samples were obtained at the end of the experiment.

**Table 4:** Biochemical profile parameters of SHR and WKY rats.

Parameter	Range	Strain	WKY	SHR			
		Group	Control Normotensive	Control (−)	Low Dose	High Dose	Control (+)
		Administration					
			Water	Water	LPDP (50 mg/kg BW)	LPDP (500 mg/kg BW)	Lisinopril (10 mg/kg BW)
Serum glucose	70–208	Unit	127 ± 13 ^ab^	134 ± 6 ^ab^	127 ± 10 ^ab^	149 ± 8 ^ab^	93 ± 9 ^b^
		mg/dL					
Serum urea	12–48	mg/dL	56 ± 5 ^a^	46 ± 4 ^a^	44 ± 4 ^a^	46 ± 6 ^a^	46 ± 4 ^a^
Serum creatinine	0.2–0.8	mg/dL	1.1 ± 0 ^a^	1.1 ± 0.09 ^a^	1.5 ± 0.2 ^a^	1.1 ± 0.1 ^a^	1.1 ± 0.1 ^a^
Total cholesterol	37–85	mg/dL	75 ± 2 ^a^	43 ± 2 ^b^	38 ± 2 ^b^	41 ± 1 ^b^	43 ± 3 ^b^
Cholesterol HDL	10–42	mg/dL	18 ± 0.8 ^a^	11 ± 1.2 ^ab^	10 ± 0.7 ^b^	11 ± 0.7 ^ab^	10 ± 2.0 ^ab^
Cholesterol LDL	20–50	mg/dL	46 ± 2.2 ^a^	25 ± 1.02 ^b^	22 ± 0.8 ^b^	24 ± 1.5 ^b^	25 ± 1.5 ^b^
Triglycerides	20–114	mg/dL	46 ± 2 ^a^	36 ± 3 ^a^	34 ± 2 ^a^	36 ± 3 ^a^	38 ± 5 ^a^
Direct bilirubin	0.03–0.2	mg/dL	0.4 ± 0.07 ^a^	0.2 ± 0.03 ^b^	0.2 ±0.02 ^b^	0.2 ± 0.03 ^b^	0.2 ± 0.02 ^b^
Indirect bilirubin	0.01–0.12	mg/dL	0.07 ± 0.02 ^a^	0.2 ± 0.05 ^a^	0.2 ± 0.01 ^a^	0.2 ± 0.03 ^a^	0.2 ± 0.07 ^a^
Total bilirubin	0.05–0.4	mg/dL	0.5 ± 0.06 ^a^	0.4 ± 0.05 ^a^	0.3 ± 0.03 ^a^	0.4 ± 0.04 ^a^	0.4 ± 0.1 ^a^
Aspartate aminotransferase (TGO-AST)	74–343	U/L	770 ± 210 ^ab^	960 ± 150 ^ab^	640 ± 100 ^b^	1050 ± 230 ^ab^	1420 ± 320 ^a^
Alanine aminotransferase (TGP-ALT)	18–145	U/L	200 ± 200 ^a^	400 ± 200 ^a^	170 ± 180 ^a^	280 ± 100 ^a^	1400 ± 250 ^b^
Glycated hemoglobin (HbA1c)	3–5.4	%	6 ± 0.4 ^a^	6 ± 0.25 ^a^	6 ± 0.33 ^a^	6 ± 0.35 ^a^	5 ± 0.4 ^a^
Serum sodium	142–151	mmol/L	136 ± 1 ^a^	136 ± 2 ^a^	139 ± 1 ^a^	136 ± 1 ^a^	138 ± 1 ^a^
Serum potassium	3.82–5.55	mmol/L	3.9 ± 0.1 ^a^	3.6 ± 0.2 ^a^	3.7 ± 0.1 ^a^	3.7± 0. 2 ^a^	3.9 ± 0.05 ^a^
Serum chlorine	100–106	mmol/L	99 ± 3 ^a^	101 ± 2 ^a^	100 ± 1 ^a^	99 ± 2 ^a^	100 ± 2 ^a^

The values shown represent the median of each group ± SEM (*n* = 5). Different letters displayed as superscripts in each row represent significant difference in each parameter between groups, analyzed with Kruskal–Wallis and Dunn as *post hoc* tests according to the normality test, (*p* < 0.05). The samples were obtained at the end of the experiment.

## Data Availability

The data presented in this study are available upon request from the corresponding author.

## Supplementary Materials

The following supporting information can be downloaded at: https://www.mdpi.com/article/10.3390/molecules31142427/s1, Figure S1: Maximum likelihood phylogenetic tree based of the 16 s rRNA sequences of strain 2501 and the type strains of the *Lacticaseibacillus* species. The nucleotide substitution model was GTR + R according to akaike criterion. Bootstrap values > 50% after 1000 pseudoreplicates are shown at nodes.

## Abbreviations

The following abbreviations are used in this manuscript:

ACE	Angiotensin Converting Enzyme
RAAS	Renin–Angiotensine–Aldosterone system
PUFA	Polyunsaturated Fatty Acid
ALA	Alpha-Linolenic Acid
DHA	Docosahexaenoic Acid
EPA	Eicosapentanoic acid
LAB	Lactic Acid Bacteria
ATP	Adenosine Triphosphate
LPDP	*Lacticaseibacillus paracasei*-derived products
BSC	Benzenesulfonyl Chloride
HHL	Hippuryl-Histidyl-Leucine
ANOVA	Analysis of Variance
SEM	Standard Error of the Mean
PCR	Polymerase Chain Reaction
HPLC-QToF-MS	High-Pressure Liquid Chromatographic–Quadrupole Time of Flight–Mass Spectrometry
DA	Dalton
TM	Transmembrane
LR	Linker region
Cyt	Cytosolic
BW	Body Weight
MCV	Mean Corpuscular Volumen
HCM	Hemoglobin Corpuscular Mean
MCHC	Mean Corpuscular Hemoglobin Concentration
HDL	High-Density Lipoprotein
LDL	Low-Density Lipoprotein
TGP-ALT	Alanine Aminotransferase
TGO-AST	Aspartate Aminotransferase
HbA1c	Glycated Hemoglobin
SHR	Spontaneously Hypertensive Rats
WKY	Wistar Kyoto Rats
