# Supplementary material for: Lactobacilli-Fermented Chia Seeds as a Potential Anti-Hypertensive Agent
Source: Molecules. 2026 Jul 10;31(14):2427. doi: 10.3390/molecules31142427 (PMC13414342; doi:10.3390/molecules31142427)
Supplement: Supplementary file 1 [file molecules-31-02427-s001.zip › molecules-4357886-supplementary.pdf]

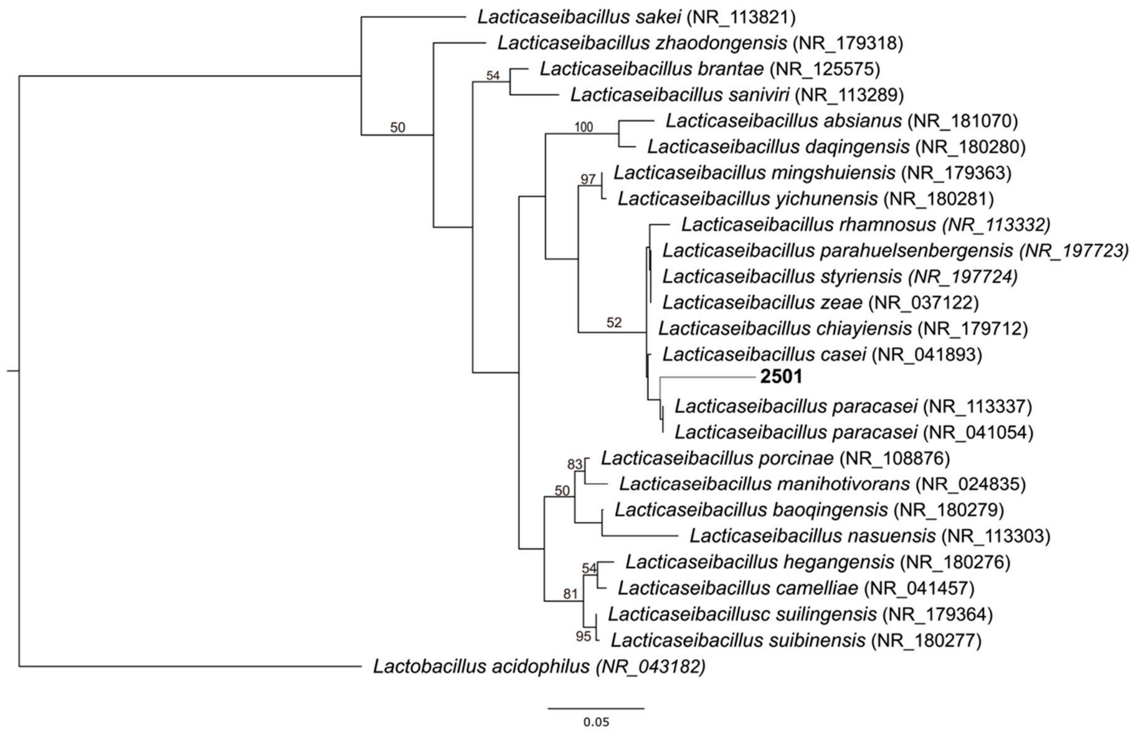

Figure S1. Maximum likelihood phylogenetic tree based of the 16s rRNA sequences of strain 2501 and the type strains of the *Lacticaseibacillus species*. The nucleotide substitution model was GTR + R according to akaike criterion. Bootstrap values > 50% after 1,000 pseudoreplicates are shown at nodes.
